# Assessing Autophagy in Microglia: A Two-Step Model to Determine Autophagosome Formation, Degradation, and Net Turnover

**DOI:** 10.3389/fimmu.2020.620602

**Published:** 2021-01-29

**Authors:** Ainhoa Plaza-Zabala, Virginia Sierra-Torre, Amanda Sierra

**Affiliations:** ^1^ Department of Pharmacology, University of the Basque Country UPV/EHU, Leioa, Spain; ^2^ Glial Cell Biology Lab, Achucarro Basque Center for Neuroscience, Leioa, Spain; ^3^ Department of Neuroscience, University of the Basque Country UPV/EHU, Leioa, Spain; ^4^ Ikerbasque Foundation, Bilbao, Spain

**Keywords:** autophagy, autophagosome, formation, degradation, LC3, microglia

## Abstract

Autophagy is a complex process that encompasses the enclosure of cytoplasmic debris or dysfunctional organelles in membranous vesicles, the autophagosomes, for their elimination in the lysosomes. Autophagy is increasingly recognized as a critical process in macrophages, including microglia, as it finely regulates innate immune functions such as inflammation. A gold-standard method to assess its induction is the analysis of the autophagic flux using as a surrogate the expression of the microtubule-associated light chain protein 3 conjugated to phosphatidylethanolamine (LC3-II) by Western blot, in the presence of lysosomal inhibitors. Therefore, the current definition of autophagy flux actually puts the focus on the degradation stage of autophagy. In contrast, the most important autophagy controlling genes that have been identified in the last few years in fact target early stages of autophagosome formation. From a biological standpoint is therefore conceivable that autophagosome formation and degradation are independently regulated and we argue that both stages need to be systematically analyzed. Here, we propose a simple two-step model to understand changes in autophagosome formation and degradation using data from conventional LC3-II Western blot, and test it using two models of autophagy modulation in cultured microglia: rapamycin and the ULK1/2 inhibitor, MRT68921. Our two-step model will help to unravel the effect of genetic, pharmacological, and environmental manipulations on both formation and degradation of autophagosomes, contributing to dissect out the role of autophagy in physiology and pathology in microglia as well as other cell types.

## Introduction

Autophagy is a complex phenomenon dedicated to eliminate intracellular debris, from protein aggregates to dysfunctional organelles, and is thus essential to maintain cell fitness (1, 2). In the brain, initial studies focused on its major role in neuronal survival (3, 4), but more recent evidence suggests that autophagy also controls health and function of other brain cell types, including microglia, the brain macrophages (1, 5). Autophagy controls several processes in microglia, including metabolic fitness (6), inflammation, phagocytosis of amyloid beta in rodent models of Alzheimer’s disease (7), degradation of extracellular beta-amyloid fibrils (8) and synuclein (9), myelin phagocytosis in acute experimental encephalomyelitis (10), as well as synaptic pruning and social behavior in mice (11). Overall, autophagy is emerging as a major controller of immune cell function, regulating innate and adaptive immune responses (12).

**Figure 1:**
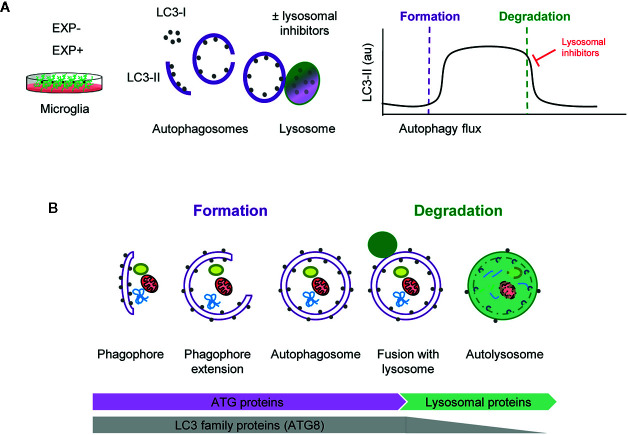
Estimation of autophagy flux variations using LC3 turnover assay. **(A)** Total protein homogenates obtained from microglia under control (EXP^-^) and experimental conditions (EXP^+^) are analyzed by Western Blot to evaluate LC3 levels in the presence and absence of lysosomal inhibitors. When autophagy is activated, LC3-I (soluble form) is lipidated to the phophatidylethanolamine of the nascent phagophore forming LC3-II (membrane-bound form). LC3-II accumulates along the extension of the autophagic vacuoles as it closes and is used as an estimate of the number of autophagosomes. Upon fusion with lysosomes, LC3-II levels decrease due to the degradation of the inner autophagosomal membrane simultaneously with the luminal cargo. In the presence of lysosomal inhibitors, no degradation occurs and LC3-II levels are maintained. The subtraction of LC3-II quantities in the presence and absence of lysosomal inhibitors provides an estimate of the autophagosomes that have been degraded during the experimental period of time. **(B)** Early stages of autophagy, which lead to the *de novo* formation of autophagosomes, are mainly regulated by ATG proteins. The LC3 family of proteins (ATG8) participate in the formation of autophagosomes and progressively disappear after lysosomal fusion and cargo degradation in autolysosomes. Late stages of autophagy depend on the functionality of lysosomal proteins and enzymes.

Assessing autophagy is complicated and current guidelines recommend using several complementary methods (13). Nonetheless, the gold standard remains the analysis of the autophagic flux using LC3 (microtubule-associated light chain protein 3). During autophagy, cytosolic LC3 (LC3-I) is conjugated to phosphatidylethanolamine and recruited to the nascent phagophore membranes (LC3-II). The phagophore then encloses cytosolic material or organelles forming a double-membrane autophagosome, which is then redirected towards the lysosome for its enzymatic degradation. The autophagic flux is calculated as the differential amount of LC3-II in the presence/absence of lysosomal inhibitors, such as bafilomycin or chloroquine, among others. As lysosomal degradation is inhibited autophagosomes accumulate and, therefore, the change in LC3-II expression informs about the autophagosomes that would have been degraded, ergo, it is a measure of autophagosome degradation. However, LC3-II Western blot raw data contains information about both autophagosome formation and degradation (**Figure 1A**).

Importantly, formation and degradation are regulated by concerted but independent mechanisms: most autophagic-regulatory genes are involved in the early stages of autophagy, as is the case of the ATG family encoding proteins that are mainly involved in autophagosome formation and maturation (1, 14). In contrast, autophagosome degradation largely depends on lysosomal proteins and enzymes (**Figure 1B**). Therefore, both early and late stages of autophagy should be systematically analyzed to understand the autophagosome turnover in any given condition.

## Methods

### Cell Culture

The murine microglial BV2 cell line and primary microglia were used to test autophagy modulating compounds. BV2 microglia were grown and maintained in Dulbecco’s Modified Eagle Medium (DMEM) (Gibco) supplemented with Fetal Bovine Serum 10% (FBS, Gibco) and a mixture of antibiotics/antimycotic (1%) including, penicillin, streptomycin, and amphotericin (all from Gibco). For experiments, 1x10^6^ cells adhered to uncoated plastic plates were used. Primary microglia cultures were performed as previously described (15, 16). Postnatal day 0–1 (P0-P1) fms-EGFP mice pup brains were extracted, and the meninges were peeled off. The olfactory bulb and cerebellum were discarded, and the rest of the brain was then mechanically homogenized by careful pipetting and enzymatically digested with papain (20 U/ml, Sigma), and deoxyribonuclease (DNAse; 150 U/µl, Invitrogen) for 15 min at 37°C. The resulting cell suspension was then filtered through a 40 μm nylon cell strainer (Fisher) and transferred to a 50 ml Falcon tube quenched by 5 ml of 20% FBS (Gibco) in HBSS. Afterwards, the cell suspension was centrifuged at 200 g for 5 min, the pellet was resuspended in 1 ml DMEM (Gibco) supplemented with 10% FBS and 1% Antibiotic/Antimycotic (Gibco), and seeded in T75 Poly L-lysine-coated (15 μl/ml, Sigma) culture flasks at a density of two brains per flask. Medium was changed the day after and then every 3–4 days, always enriched with Granulocyte Macrophage Colony Stimulating Factor (5 ng/ml GM-CSF, Sigma). After confluence (at 37°C, 5% CO_2_ for approximately 14 d), microglia cells were harvested by shaking at 100–150 rpm, 37°C, 4 h. Isolated cells were counted and plated at a density of 2x10^6^ cells/well on poly-l-lysine-coated plastic plates. BV2 and primary microglia were allowed to settle for at least 24 h before experiments.

### Drug Treatments

BV2 microglia were treated with rapamycin 100 nM (Fisher Scientific) for 6 h in the presence and absence of bafilomycin 100 nM (SelleckChem) for autophagy induction. Primary microglia were treated with the autophagy inhibitor MRT68921 1, 10, or 30 µM (Sigma) for 3 or 6 h with or without bafilomycin 100 nM (SelleckChem).

### Protein Extraction and Western Blot

Microglia were directly lysed in plastic plates with RIPA buffer containing protease inhibitor cocktail (100x) (ThermoFisher). The cell suspension was then sonicated for 5s and centrifuged (10,000 g, 10 min) to obtain solubilized protein in the supernatant. Sample protein content was quantified in triplicates by BCA (Bicinchoninic Acid) assay kit (ThermoFisher) at 590 nm using a microplate reader (Synergy HT, BioTek). β-mercaptoethanol denatured proteins (15–20 ug) were loaded onto 14% Tris-glycine polyacrylamide gels (ThermoFisher) and run at 120V for 90min. Protein samples were then blotted to nitrocellulose membranes (0.45 µm pore size) (ThermoFisher) at 200 mA for 90 min or using the Trans-Blot Turbo Mini Nitrocellulose Transfer Pack (Bio-Rad). Transfer efficiency was verified by Ponceau S (Sigma) staining. For immunoblotting, membranes were rinsed in Tris Buffered Saline containing 0.1% Tween 20 (Sigma) (TBS-T) and then blocked for 1 h in TBS-T containing 5% powder milk. Membranes were afterwards incubated with rabbit primary antibody to LC3 (1:3,000, NB100-2220, Novus Biologicals), and mouse primary antibody to β-actin (1:5,000, Sigma), in TBS-T containing 4% Bovine Serum Albumin (BSA) overnight (4°C, shaker). Next day, membranes were rinsed and incubated with Horseradish Peroxidase (HRP) conjugated anti-rabbit (1:5,000) and anti-mouse (1:5,000) secondary antibodies (Cell Signaling) for the rapamycin blot or with the fluorescent StarBright Blue 700 anti-mouse (1:5,000) and StarBright Blue 700 anti-rabbit (1:5,000) secondary antibodies (Bio-Rad) for the MRT68921 blots in TBS-T containing 5% powder milk. After rinsing membranes, protein was visualized by Enhanced ChemiLuminescence (ECL) using Supersignal West Femto Maximum Sensitivity Substrate (ThermoFisher) for the rapamycin blot or by immunofluorescence for the MRT68921 blots, in a ChemiDoc imaging system (BioRad). Band intensity was quantified using the Gel Analyzer method of Fiji software.

### Statistics

Statistical analysis was performed with SigmaPlot. Normality and homoscedasticity were assessed prior to analysis. Raw LC3 data was initially analyzed by two-way ANOVA, but since an interaction between treatment (rapamycin, MRT68921) and bafilomycin was found, the global effect of the treatment was subsequently analyzed by one-way ANOVA. In addition, flux, and formation and degradation rates were analyzed by one-tail Student t-test (**Figures 5A, B**) or by one-way ANOVA followed by a Holm-Sidak posthoc test (**Figure 5C**). Formation and degradation rates were compared to one using a one-tail Student t-test. Data is shown as mean ± SEM. Only tests with p <0.05 are considered significantly different; tests with p <0.1 are reported to have a tendency.

## Modeling Autophagosome Formation and Degradation

**Figure 2:**
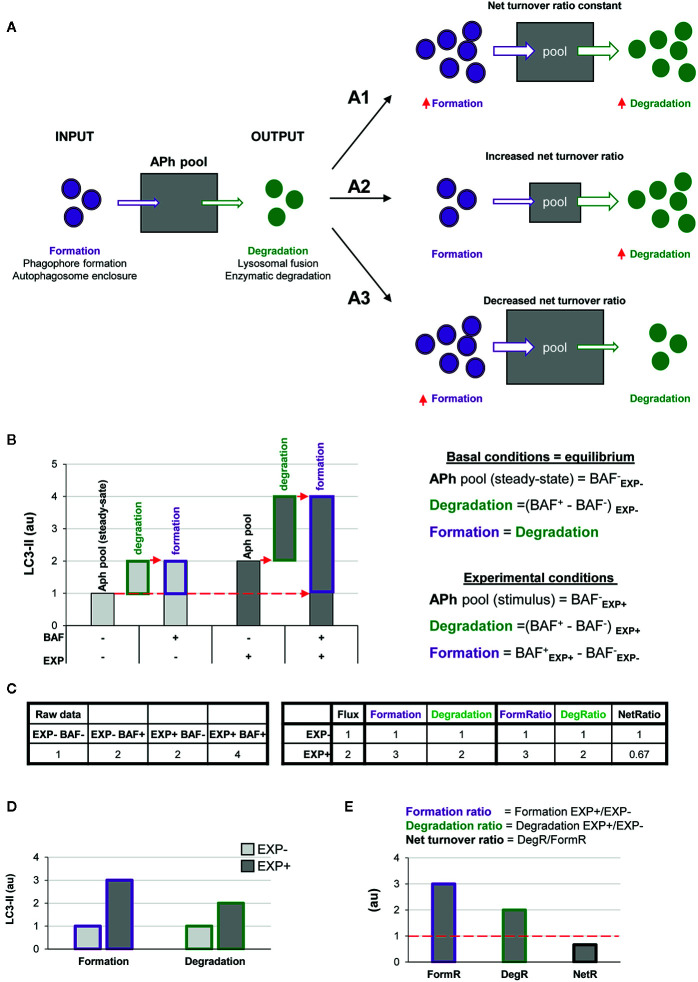
A two-step model of autophagy to analyze formation and degradation of autophagosomes. **(A)** The model represents the autophagosomes as a box with an input (autophagosome formation, purple dots) and an output (autophagosome degradation, green dots) that determines the autophagosome net turnover. A1–A3**** represent different possible scenarios with no changes (A1****), an increase (A2****) and a decrease (A3****) in the autophagosome net turnover. **(B)** Graph representing the amount of LC3-II (au, arbitrary units) in two experimental conditions representing (EXP^-^ and EXP^+^) in the presence or absence of the lysosomal inhibitor bafilomycin (BAF^-^ and BAF^+^), and the formulas used to calculate formation, degradation, and net turnover. The dotted red arrows mark the LC3-II raw data values used to calculate the formation and degradation rates and ratios. **(C)** Simulated raw LC3-II data (au) (left) used to calculate the formation and degradation rates and ratios (right) used in the graphs shown in **(B, D**, **E)**. **(D**, **E)** Graphs representing the rate of change of formation, degradation and net turnover between the two experimental conditions.

Here we propose a simple two-step model to analyze autophagy, in which the net number of autophagosomes (i.e., the autophagosome pool) at any given time is treated as a black box to which there is an input (formation) and an output (degradation) (**Figure 2A**). The formation phase encompasses phagophore formation, cargo sequestration, and autophagosome closure, and the degradation phase summarizes the lysosomal fusion and the enzymatic degradation of the autophagosome contents. Nonetheless, the precise definition of formation/degradation in each experimental setup depends on the physiological process blocked by the particular lysosomal inhibitor used: fusion inhibitors, such as vinblastine, which blocks transport of autophagosomes by microtubules; protease inhibitors, such as E64d and leupeptin; or proton pump inhibitors, such as bafilomycin. This conceptual frame can be easily modeled by a simple equation in which the size of the autophagosome (APh) pool in a given time point depends on the number of autophagosomes in the steady-state (ss) plus the number of autophagosomes formed minus the autophagosomes degraded in a certain period of time:

$${\text{APh}}_{t}={\text{APH}}_{\text{ss}}+\text{APh Formation}-\text{APh Degradation}$$

The ratio between degradation and formation is the net autophagic turnover, which is a measure of the relative velocity of autophagosome formation versus degradation. A given stimulus could act proportionally both on the formation and the degradation, maintaining the size of the APh pool and resulting in a constant net turnover ratio (**Figure 2A1**). However, under some conditions, the regulation of the formation and degradation of autophagosomes may be dissociated: an increased degradation would decrease the size of the APh pool and increase the net turnover (**Figure 2A2**); and an increased formation would increase the size of the APh pool and decrease the net turnover (**Figure 2A3**). Thus, we propose that to understand the complexity of the biology underneath the autophagosome turnover we need to analyze separately formation, degradation, and the net autophagic turnover.

This analysis can be performed using the data available in conventional LC3 assays by Western blot. In this type of analysis, cells or tissue from two experimental conditions (EXP^-^: control and EXP^+^: experimental stimulus) are incubated in the presence or absence of lysosomal inhibitors such as bafilomycin (BAF^-^ and BAF^+^) for a certain period of time. Protein from these four conditions is extracted and the expression of LC3-II is analyzed by Western blot and, ideally, normalized to reference proteins such as actin (13). In addition, the complementary normalization of LC3-II to LC3-I may facilitate the understanding of the full picture of the autophagy response (13).

In the basal condition (EXP^-^), the amount of LC3-II in the absence of lysosomal inhibitors (BAF^-^) represents the APh pool in the steady state, analogous to taking a snapshot of the autophagic process (**Figure 2B**). The difference between the amount of LC3-II in cells incubated with and without lysosomal inhibitors (BAF^+^ – BAF^-^) in the basal condition represents the autophagosomes that have disappeared (i.e., the degradation phase), which is what is conventionally called autophagic flux. To calculate the autophagosomes that have formed, our model stems from the assumption that in the basal condition autophagy is at an equilibrium because formation and degradation occur at the same speed:

$$\begin{array}{c} \text{Basal condition}:\\ \text{APh Formation}=\text{APh Degradation}\\ {\text{APh}}_{\text{equilibrium}}={\text{APh}}_{\text{ss}} \end{array}$$

Thus, in the basal condition (EXP^-^) the autophagosomes that have formed are identical to the autophagosomes that have degraded, and thus are also represented by the amount of LC3-II with and without lysosomal inhibitors (BAF^+^ – BAF^-^) (**Figures 2B, C**).

In the experimental condition (EXP^+^), the amount of LC3-II in the absence of lysosomal inhibitors (BAF^-^ in EXP^+^) represents the size of the APh pool under the stimulus. Again, degradation can be calculated as the difference in LC3-II with and without lysosomal inhibitors [(BAF^+^ in EXP^+^) – (BAF^-^ in EXP^+^)]. Formation can be calculated as the difference between the amount of LC3-II in the presence of lysosomal inhibitors minus the size of the initial APh pool in steady-state conditions [(BAF^+^ in EXP^+^) - (BAF^-^ in EXP^-^)] (**Figures 2B, C**). This procedure allows us to calculate the formation and degradation of autophagosomes in control and experimental conditions (**Figure 2D**). To then compare whether the stimulus acts proportionally in both formation and degradation, we can calculate the ratio between experimental and basal conditions (EXP^+^/EXP^-^) for both formation and degradation (**Figure 2E**). Finally, to compare the relative magnitude of degradation compared to formation, we can calculate the ratio between both as the net turnover ratio (**Figure 2E**), which has a value of one in basal conditions, because autophagosome formation and degradation occur at the same rate (red dotted line in **Figure 2E**).

## Dissecting Out Autophagosome Formation and Degradation

**Figure 3:**
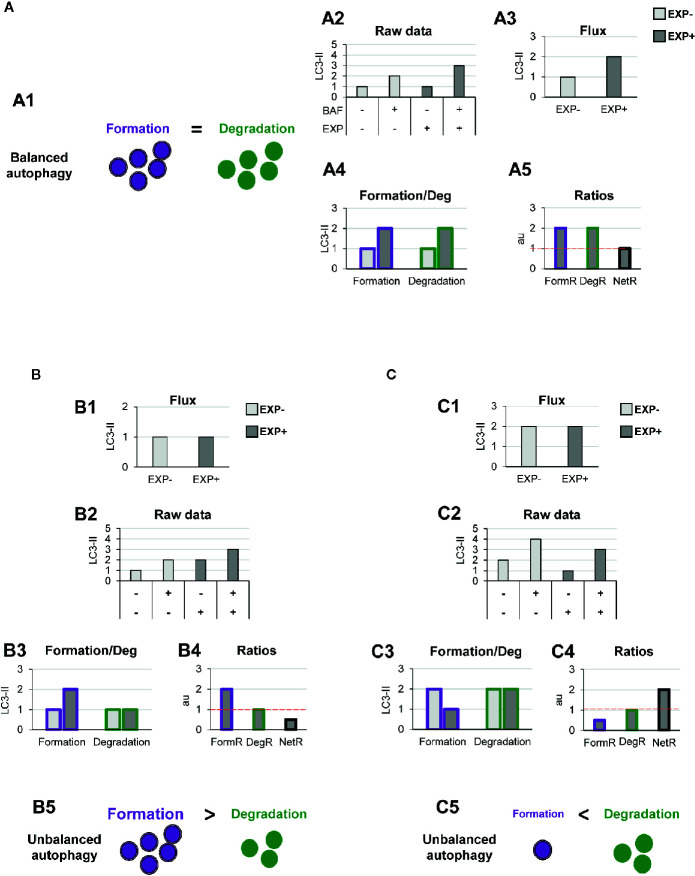
Theoretical examples of variations in the formation of autophagosomes that lead to balanced or unbalanced autophagy. **(A)** Example of a balanced flux with proportional increase in autophagosome formation and degradation. The model of balanced flux with equal formation (purple dots) and degradation (green dots) (A1), the raw LC3-II/actin Western blot data (A2), the conventional autophagy flux (A3), the formation and degradation rates (A4), and the formation, degradation, and net ratios (A5) are shown. The red dotted line represents the threshold of one to determine a significant change (over 1, basal conditions) in the formation, degradation and net turnover ratios. **(B**, **C)** Show examples with similar conventional flux (B1, C1), which are in fact derived from dissimilar raw LC3-II/actin Western blot data (B2, C2). In **(B)** Our model would reveal increased autophagosome formation rate and no changes in degradation rate (B3), leading to an increased formation ratio and reduced net ratio (B4), and an unbalanced autophagy (B5). In contrast, in **(C)** our model would reveal decreased autophagosome formation rate and no changes in degradation rate (C3), leading to an increased formation ratio and reduced net ratio (C4), and an unbalanced autophagy (C5).

This model allows us to discriminate and quantify different potential biological scenarios that may affect autophagosome formation, degradation, or both. For instance, a typical autophagic stimulus would be expected to proportionally increase autophagosome formation and degradation, maintaining a balanced autophagy (**Figure 3A1**). Examples of this scenario are treatment with the well-known autophagy activator rapamycin, an inhibitor of the mTORC1 complex (Mechanistic Target Of Rapamycin Complex 1) (17); or activation of the transcription factor-EB (TFEB), which coordinately regulates the biogenesis of autophagosomes and lysosomes (18), maintaining the equilibrium between formation and degradation.

To exemplify this scenario, we simulated raw LC3-II Western blot data from a canonical autophagy stimulus (**Figure 3A2**), and from here we calculated the classic autophagy flux, showing the expected increase (**Figure 3A3**). We then applied our model to the raw data and observed that the canonical autophagic stimulus increased both formation and degradation (**Figure 3A4**). Importantly, both formation and degradation ratios were similar and, as a result, the net autophagy ratio was constant (**Figure 3A5**), implying a maintenance of the net autophagic turnover but at a higher rate/velocity, that could be possibly maintained in the long term.

In contrast, there are other biological scenarios that are not so easily discriminated using the conventional calculation of the autophagy flux (**Figures 3B, C**). Examples of these scenarios are situations in which autophagosome formation is increased (**Figure 3B**) or decreased (**Figure 3C**), without concomitantly affecting degradation. For instance, overexpression of ATG proteins or accumulation of intracellular debris would lead to increased autophagosome formation. But if lysosomal efficiency (i.e., degradation) is not proportionally increased, autophagosomes will stall in the lysosomes without degrading the autophagic cargo, leading to a decreased net turnover ratio and increased autophagosome pool. This effect has been for example observed in cells that overexpress Atg5 but whose lysosomal function is compromised (19). In this case, calculation of the autophagy flux would not reveal any changes (**Figures 3B1, C1**), although the raw LC3-II data is evidently different (**Figures 3B2, C2**). Our model would help to quantify the specific effect on formation (**Figures 3B3, C3**), and the alteration of the net autophagy ratio (**Figures 3B4, C4**), revealing an unbalanced autophagy (**Figures 3B5, C5**), and a potentially catastrophic situation for the cell that could not possibly be maintained over time.

**Figure 4:**
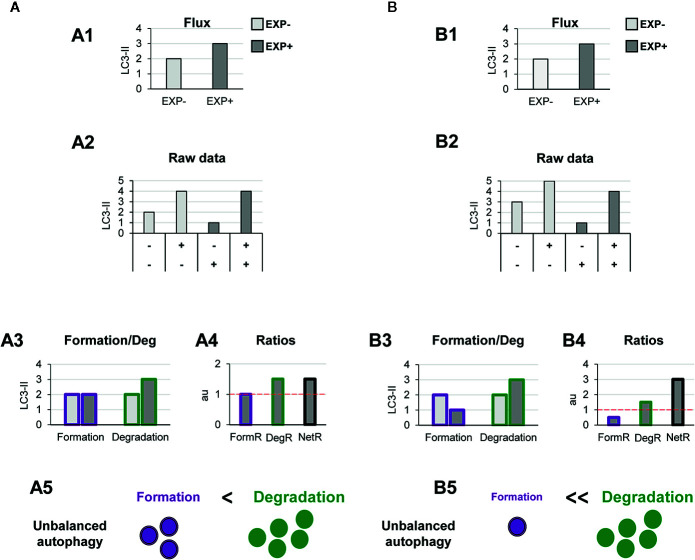
Theoretical examples of increased autophagosome degradation that lead to unbalanced autophagy. **(A, B)** show examples with similar conventional flux (A1, B1), derived from apparently similar raw LC3-II/actin Western blot data (A2, B2). In **(A)** our model would reveal an increased autophagosome degradation rate and no changes in the formation rate (A3), leading to an increased degradation ratio and net ratio (A4), and an unbalanced autophagy (A5). In contrast, in **(B)** our model would reveal decreased autophagosome formation rate but increased degradation (B3), leading to decreased formation ratio, increased degradation ratio, and a strong increase in the net ratio (C4), ultimately resulting in a highly unbalanced autophagy (B5).

**Figure 5:**
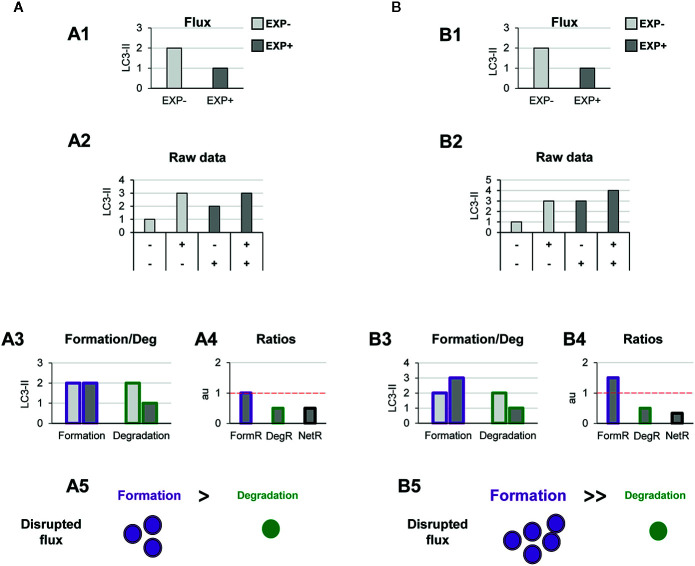
Theoretical examples of decreased autophagosome degradation that lead to unbalanced autophagy. **(A, B)** show examples with similar conventional flux (A1, B1), derived from apparently similar raw LC3-II/actin Western blot data (A2, B2). In **(A)** our model would reveal a decreased autophagosome degradation rate and no changes in the formation rate (A3), leading to a reduced degradation ratio and net ratio (A4), and an unbalanced autophagy (A5). In contrast, in **(B)** our model would reveal increased autophagosome formation rate but decreased degradation (B3), leading to increased formation ratio, reduced degradation ratio, and a strong reduction in the net ratio (C4), ultimately resulting in a highly unbalanced autophagy (B5).

Other biological scenarios that cannot be discriminated using conventional analysis of the autophagy flux are shown in **Figures 4**, **5**. Some stimuli may selectively increase autophagosome degradation without affecting their formation coordinately (**Figure 4A**), or even reducing it (**Figure 4B**). For example, enhanced lysosomal biogenesis or lysosomal enzymes efficiency might lead to increased autophagosome degradation, resulting in an increased net turnover ratio and reduced autophagosome pool size. This imbalance has been reported in mice genetically deficient for the cathepsin inhibitor cystatin B, which exhibit enhanced lysosomal proteolysis (20). Whereas in this case the calculation of the autophagy flux would suggest an enhanced autophagy, our model would reveal the imbalance between formation and degradation, suggesting that in fact cellular debris would not be removed any faster from the cytoplasm.

Another scenario in which our model may prove useful is one where autophagosome degradation is reduced but formation is maintained (**Figure 5A**) or even increased (**Figure 5B**). An example of this scenario is a pathological condition where dysfunctional organelles accumulate and the cell tries to enclose them in autophagosomes but lysosomal functionality is compromised, for instance because lysosomes are defective or engaged in other degradation pathways such as phagocytosis or endocytosis. This effect could be observed in Parkinson’s disease (PD) dopaminergic neurons, which contain LC3-positive Lewy bodies, and have stalled autophagosomes, and lysosomal depletion (21). This complex effect cannot be fully understood by simply analyzing the reduction in the autophagy flux but would be instead clearly described by our two-step model.

## Testing the Model *in Vitro*

We have directly tested our model with experimental data using two well-characterized autophagy modulators: the autophagy inducer rapamycin, which inhibits mTORC1 (22); and the autophagy inhibitor MRT68921, which blocks ULK1/2 (unc-51-like kinases 1/2) (22, 23). Both mTORC1 and ULK1/2 are early checkpoints of canonical autophagy: mTORC1 transduces signals from energy and damage sensors and is inhibited under stressful situations, releasing ULK1/2 (unc-51-like kinase 1/2) by a series of phosphorylation and dephosphorylation events to initiate the autophagy cascade (1, 14). As a cell model we used cultures of microglia (BV2 cells or primary cultures) and analyzed the amount of LC3-II by Western blot as a measurement of the size of the autophagosome pool.

**Figure 6:**
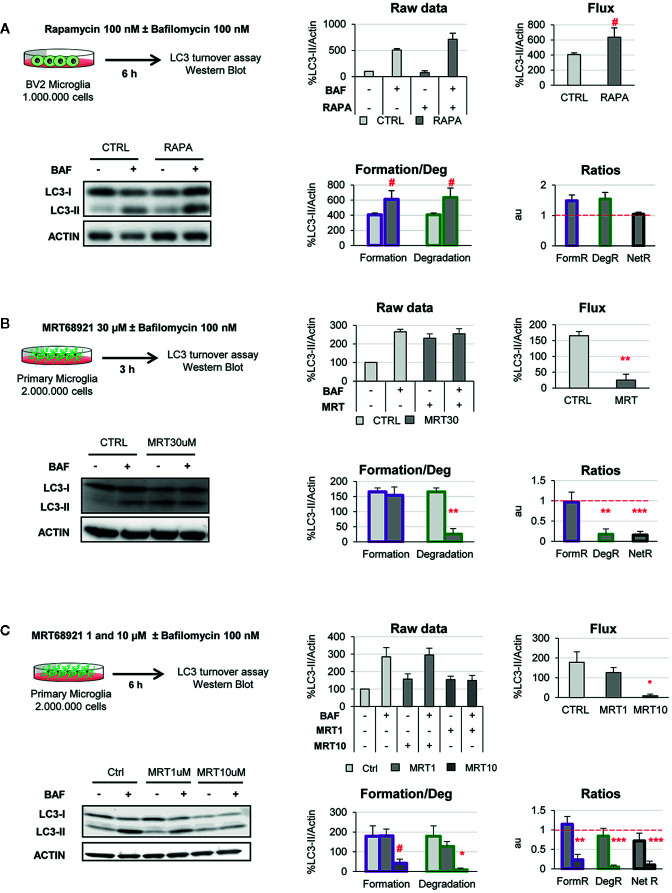
Validation of the two-step model with autophagy modulating compounds. **(A)** Autophagy induction assessed after treatment with rapamycin (100 nM, 6 h) in the presence and absence of Bafilomycin (100 nM) in the BV2 microglia cell line. A representative blot, the raw data obtained, and the calculations of flux, autophagosome formation and degradation, and net turnover ratios are shown. Data is presented as % over control (LC3-II/actin). **(B, C)** Autophagy inhibition assessed after treatment with MRT68921 (30 μM, 3 h in **(B)**; 1 and 10 μM, 6 h in **(C)** in the presence and absence of Bafilomycin (100 nM) in mouse primary microglia. A representative blot, the raw data obtained, and the calculations of flux, autophagosome formation and degradation, and net turnover ratios are shown. Data is presented as % over control (LC3-II/actin). Data represent mean ± SEM of 3 independent experiments. ^#^represents p < 0.1, *represents p < 0.05 and ** represents p < 0.01 by one tailed Student t-test **(A, B)**, or Holm-Sidak after a significant effect of the treatment was found with 1-way ANOVA **(C)**.

In BV2 microglia rapamycin (6 h, 100 nM) showed the expected response and a tendency to increased LC3-II flux (**Figure 6A**). In addition, our model uncovered a parallel increase in formation and degradation of autophagosomes, resulting in a constant size of the APh pool and no changes in the net autophagosome turnover. Thus, rapamycin allowed the maintenance of the equilibrium between formation and degradation (**Figure 6A**), indicating a sustained autophagy that the cell can maintain over time.

On the other hand, MRT68921 (3 h, 30 μM) resulted in the expected decrease in the LC3-II flux in primary microglia (**Figure 6B**). However, analysis with our model revealed that only degradation was reduced whereas autophagosome formation remained constant (**Figure 6B**). This data is in apparent contradiction with the described role of MRT in blocking the autophagy pre-initiation complex (22, 23). To address this discrepancy, we used a second paradigm of MRT68921 with a longer treatment and lower dosage (6 h, 1–10 μM; **Figure 6C**), and observed that the upstream effect of inhibition of autophagosome formation with MRT 10µM translated into a similar decrease in degradation (**Figure 6C**). Therefore, our model proves useful to discriminate the effect of experimental manipulations on the formation and/or degradation of autophagosomes.

## Future Directions

Autophagy is a complex multi-step phenomenon and its assessment is a complicated task that requires using complimentary methods, as most current guidelines recommend (13, 24). Visualization of double-membrane autophagosomes by transmission electron microscopy, live imaging of LC3 acidification using ratiometric analysis of fluorophores, or analysis of substrate degradation should corroborate the data obtained by analysis of LC3-II expression as a proxy for autophagosome formation and degradation. It is also important to note that autophagy is a time-dependent process and, as such, its dynamics should be assessed over time (25). In addition, LC3-II immunoblotting assays have several limitations, such as the reference protein used to normalize LC3-II values, the timing and concentration of the lysosomal inhibitor used, or the intrinsic nonlinear detection of proteins by enhanced chemoluminescence (ECL) (26). The most widely used method to asses autophagy is, nonetheless, the analysis of the LC3-II flux in the presence of lysosomal inhibitors. However, the complexities associated to interpreting LC3-II flux have been thoroughly pointed out before, in the quest for an optimal “autophagomometer” (26). One of the key points is that autophagosomes formation and degradation are spatially and temporally dissociated (27) and that therefore they need to be assessed independently.

To address this issue, we here propose a simple conceptual frame to help interpreting LC3-II flux experiments. Our two-step model conceives the steady-state levels of LC3-II as an indirect measure of the pool of autophagosomes present when the snap-shot is taken. Assuming that in the basal condition the cells or tissue of interest are in some sort of equilibrium, the amount of autophagosomes formed and degraded should be roughly the same. Thus, the autophagosome pool can be treated as a black box to which the input (formation) and output (degradation) are identical, and can be estimated as the difference between LC3-II levels in the presence and absence of lysosomal inhibitors. In the experimental condition, degradation can be similarly calculated as the difference between LC3-II levels in the presence and absence of lysosomal inhibitors (i.e., the conventional LC3-II flux). In addition, we propose that the formation of autophagosomes in the experimental condition can be estimated by subtracting the steady-state autophagosome pool to the autophagosomes that have accumulated in the presence of lysosomal inhibitors. This model allows us to dissect out the effects of the experimental conditions to autophagosome formation and degradation. In addition, it also allows us to understand the net changes in the size of the autophagosomal pool that are the result of maintaining (or not) the net turnover ratio at equilibrium.

We have tested the two-step model using pharmacological autophagy modulators such as the autophagy inducer rapamycin and the autophagy inhibitor MRT68921 in microglia. As expected, rapamycin enhanced autophagy flux increasing both autophagosome formation and degradation at the concentration (100 nM) and time point (6 h) tested. However, the autophagy inhibitor MRT68921 exhibited concentration and time-dependent differential effects. At a medium concentration (10 μM) and long time-point (6 h), MRT68921 decreased both autophagosome formation and degradation, in line with the inhibitory effects described over ULK1/2 kinase activity, while no effect was observed at a lower concentration (1 μM). Nevertheless, at high concentration (30 µM) and short time-point (3h), MRT68921 selectively decreased autophagosome degradation while maintaining their formation. This was an unexpected result since MRT68921 inhibits ULK1/2 kinase, a protein mainly known for its role in autophagy initiation (28). However, ULK1/2 kinase also regulates the recruitment of other autophagy-related proteins for the productive formation of autophagosomes (23, 29). Thus, inhibition of ULK1/2 kinase activity at high concentrations and short time-points could preferentially affect autophagosome degradation activity, maintaining residual autophagy initiation activities, leading to the formation of LC3-II positive stalled phagophores and LC3-II accumulation after inhibitor treatment (23). Overall, using pharmacological modulators of autophagy, we demonstrate that our two-step model is able to accurately measure the selective changes that may occur in autophagosome formation and/or degradation in microglia after exposure of autophagy modulating stimuli.

Nonetheless, our two-step model has several limitations that should be considered. The most important one is the assumption that autophagy (formation and degradation) are at equilibrium in the basal condition. This equilibrium implies coordinated control mechanisms that would be necessary to maintain autophagy in the long term (30), but each cell type may have different regulation mechanisms under different metabolic constraints (31), and would depend on experimental conditions such as cell density. Another important point is that autophagosome formation and degradation are not independent phenomena, as assumed in our model. For instance, it is obvious that if the lysosomal pool is not a limiting factor, the degradation will directly depend on the formation. In addition, feed-back mechanisms may link excessive lysosomal degradation with a subsequent reduction in autophagosome formation (32). In spite of these limitations, our model can provide a more expanded insight into the complexity of the autophagy process than simply analyzing the autophagic flux. In summary, we here show that using the LC3 turnover assay, our two-step model helps to systematically determine changes in autophagosome formation vs degradation, the net turnover and the size of the autophagosome pool to obtain a more comprehensive understanding of autophagy.

Due to the universal nature of LC3 turnover assays, our two-step model is useful to estimate changes in autophagosome formation and degradation in virtually all mammalian cell types, including microglia. As autophagy has emerged as a regulator of a plethora of microglial functions (1) related to regulation of metabolic status, inflammation, and phagocytosis (6, 7, 10), our two-step model may provide a simple framework to understand the basic dynamics of microglial autophagy in health and disease.

## Data Availability Statement

Requests to access the datasets should be directed to AS, amanda.sierra@achucarro.org.

## Ethics Statement

The animal study was reviewed and approved by Comité de Ética en Experimentación Animal (CEEA), University of the Basque Country EHU/UPV.

## Author Contributions

AS and AP-Z conceived the idea and wrote the manuscript. VS-T performed experiments and wrote the manuscript. All authors contributed to the article and approved the submitted version.

## Funding

This work was supported by grants from the Spanish Ministry of Science and Innovation (https://www.ciencia.gob.es/) with FEDER funds (RTI2018-099267-B-I00) and a Tatiana Foundation project (P-048-FTPGB 2018) to AS. VS-T holds a predoctoral fellowship from the Basque Government.

## Conflict of Interest

The authors declare that the research was conducted in the absence of any commercial or financial relationships that could be construed as a potential conflict of interest.

## Abbreviations

APh, Autophagosome; ATG, Autophagy-related protein; BAF, Bafilomycin A1; BCA, Bicinchoninic Acid; DegR, Degradation ratio; DMEM, Dulbecco’s Modified Eagle Medium; ECL, Enhanced Chemoluminescence; EXP^-^, basal condition; EXP^+^, experimental condition; FBS, Fetal Bovine Serum; FormR, Formation ratio; GM-CSF, Granulocyte-Macrophage Colony Stimulating Factor; LC3-II, microtubule-associated light chain protein 3 conjugated to phosphatidylethanolamine; mTORC1, Mechanistic Target of Rapamycin Complex 1; NetR, Net ratio; PD, Parkinson’s disease; ss, steady-state; TBS-T, Tris Buffered Saline containing 0.1% Tween 20; TFEB, transcription factor-EB; ULK1/2, unc-51-like kinases 1/2.
