# Erratum: Assessing Autophagy in Microglia: A Two-Step Model to Determine Autophagosome Formation, Degradation, and Net Turnover

**DOI:** 10.3389/fimmu.2021.724901

**Published:** 2021-06-25

**Authors:** Frontiers Production Office

**Affiliations:** Frontiers Media SA, Lausanne, Switzerland

**Keywords:** autophagy, autophagosome, formation, degradation, LC3, microglia

Due to a production error, there was an error in affiliations 1, 2 and 3. Instead of “^1^Department of Pharmacology, Achucarro Basque Center for Neuroscience, Leioa, Spain, ^2^Glial Cell Biology Lab, Achucarro Basque Center for Neuroscience, University of the Basque Country UPV/EHU, Leioa, Spain, ^3^Department of Neuroscience, Achucarro Basque Center for Neuroscience, Leioa, Spain,” it should be “^1^Department of Pharmacology, University of the Basque Country UPV/EHU, Leioa, Spain, ^2^Glial Cell Biology Lab, Achucarro Basque Center for Neuroscience, Leioa, Spain, ^3^Department of Neuroscience, University of the Basque Country UPV/EHU, Leioa, Spain.” The publisher apologizes for this mistake.

The original version of this article has been updated.

